# Influence of ER-CR-YSGG Laser and Photodynamic Therapy on the Dentin Bond Integrity of Nano-Hydroxyapatite Containing Resin Dentin Adhesive: SEM-EDX, Micro-Raman, Micro-Tensile, and FTIR Evaluation

**DOI:** 10.3390/polym13121903

**Published:** 2021-06-08

**Authors:** Abdullah S. Aljamhan, Mohammad H. Alrefeai, Alhanouf Alhabdan, Sarah A. Alhusseini, Imran Farooq, Fahim Vohra, Mustafa Naseem, Fahad Alkhudhairy

**Affiliations:** 1 Restorative Dental Sciences Department, College of Dentistry, King Saud University, Riyadh 11545, Saudi Arabia; a.aljamhan@ksu.edu.sa (A.S.A.); malrefeai@ksu.edu.sa (M.H.A.); alhabdan@ksu.edu.sa (A.A.); salhusseini@ksu.edu.sa (S.A.A.); 2 Faculty of Dentistry, University of Toronto, Toronto, ON M5G 1G6, Canada; imran.farooq@mail.utoronto.ca; 3 Prosthetic Dental Science, College of Dentistry, King Saud University, Riyadh 11545, Saudi Arabia; fvohra@ksu.edu.sa; 4 Department of Community and Preventive Dental Sciences, Dow International Dental College, Karachi 74200, Pakistan; mustafa.naseem@duhs.edu.pk

**Keywords:** adhesive, hydroxyapatite, H_3_PO_4_, photodynamic therapy, laser

## Abstract

The study aimed to analyze the effect of the addition of nano-hydroxyapatite (nano-HA) particles on the mechanical properties of experimental adhesive (EA). Furthermore, dentin interaction of EA (without nano-HA) and EA with nano-HA (hereon referred to as HA-10%) were also investigated and equated. Methods consisting of scanning electron microscopy (SEM)–energy-dispersive X-ray spectroscopy (EDX), micro-Raman spectroscopy, micro-tensile bond strength (µTBS) test, and Fourier transform infrared (FTIR) spectroscopy were employed to study nano-HA particles shape, dentin bond strength, degree of conversion (DC), and adhesive–dentin interaction. Ninety teeth (N = 90) were collected, and pre-bonding, conditioning of dentin was performed utilizing phosphoric acid (H_3_PO_4_) etching, photodynamic therapy (PDT), and ER-CR-YSGG (ECY) laser. The teeth were set to form bonded specimens using two adhesives. Nano-HA particles were spherical-shaped, and EDX confirmed the presence of oxygen, calcium, and phosphorus. Micro-Raman spectroscopy revealed distinct phosphate and carbonate peaks for nano-HA. The µTBS test demonstrated highest values for HA-10% group on the H_3_PO_4_ conditioned dentin. The greatest DC was observed for the EA group. The addition of nano-HA-10 wt.% particles in dentin adhesive resulted in improved bond strength. The incorporation also demonstrated acceptable DC (although lower than EA group), suitable dentin interaction, and resin tag formation.

## 1. Introduction

Resin composites have been increasingly used by dental practitioners globally over the last few decades due to their superior aesthetics [1]. Concerns hovering over the possible adverse health effects due to mercury release from dental amalgams have also steered intensification in the usage of dental composites [2]. Conversely, resin composites have questionable longevity (mean replacement period: 5.7 years), which is primarily due to the development of secondary caries and restoration fractures [3]. Dentin adhesives (DA) play a crucial role in governing the longevity of composite restorations, but adhesion is directly reliant on the developed polymer’s quality [4]. DA can stimulate bond formation amongst the conditioned dentin surface and composite resin by cross-linking primer (hydrophilic in nature) with resin (hydrophobic in nature) [5]. The DA establishes a direct connection with the tooth surface; therefore, its inclusion inside the adhesive could be more beneficial than its incorporation in the composite resin [6]. Researchers have incorporated various nano-inorganic fillers inside the DA, as they expand mechanical properties, remineralization competence, and the bond strength of dental resin composites [6,7,8].

Hydroxyapatite (HA) minerals are natural compositional constituents of human hard tissues [9]. HA is a slowly resorbing, non-toxic, bioactive, and biocompatible material [10]. The presence of HA in a material promotes the biomimetic mineralization of dental tissues [11]. Among nanomaterials, the nano-HA particles have a similar crystal structure as that of the apatite present in the hard tissues of humans [12] and have been successfully used for various medical applications related to bone tissue engineering [13]. In dentistry, nano-HA particles have been used successfully as an implant coating material, bone grafting material, management of dentin hypersensitivity, and as nano-inorganic fillers inside DA [14]. Previously, Sadat-Shogai et al. demonstrated that the inclusion of HA particles enhances composite resin’s bond strength and decreases the hybrid layer’s degradation [15]. Certain other studies in the literature have also reported similar findings and revealed that the incorporation of nano-HA particles improves the mechanical properties of the adhesive [6,8]. Another advantage of adding nano-HA particles in the adhesive is that nano-sized filler particles do not restrict curing light’s penetration ability, which is required to convert monomers into polymers, as opposed to micro-sized particles [16].

Treatment of the dentin by various methods could enhance the longevity of the adhesive–dentin bond and fiber posts [17,18]. These methods include the conditioning of dentin with photodynamic therapy (PDT) and ER-CR-YSGG (ECY) laser [17,18]. PDT is an antimicrobial approach of conditioning, based on the chemical combination of a low-intensity laser of suitable wavelength and a photosensitizing agent [18]. A previous study has suggested that it can have a positive impact on the bond strength of dentin [18]. ECY laser usually works on a wavelength of 2780 nm and removes the dentinal smear layer [19]. Moreover, dentin conditioning of ECY laser opens up dentinal tubules that is ideal for the better retention of adhesive restorative materials [19]. In our study, we also utilized different dentin conditioning methods and probed various mechanical properties of the adhesives.

Researchers have previously investigated the impact of the addition of filler particles in the adhesive by developing their own experimental adhesive (EA) (instead of commercially acquiring them) and demonstrated that EAs are good alternatives to their commercial counterparts [20,21,22]. These EAs can have different compositions; however, essential ingredients (discussed in Section 2.1) are not changed. Considering the benefits of nano-HA particles, we decided to integrate them in our experimental adhesive (EA), as their incorporation inside EA could reinforce the adhesive’s mechanical properties. Adding the nano-HA to a commercially available adhesive may have not allowed an accurate assessment of the influence of nano-HA, as the contents of commercially available adhesive are not completely and accurately known, and the outcomes shown will not necessarily reflect the effect of nano-HA only. Therefore, our study was aimed at developing and characterizing an ethanol-based EA containing nano-HA particles using techniques such as scanning electron microscopy (SEM)–energy-dispersive X-ray (EDX) spectroscopy, micro-Raman spectroscopy, micro-tensile bond strength (μTBS) testing, Fourier transform infrared (FTIR) spectroscopy, and degree of conversion (DC) analysis. It was hypothesized that the insertion of nano-HA particles in the EA would enhance its bond strength, durability, and dentin interaction.

## 2. Materials and Methods

The study was performed in accordance with the declaration of Helsinki ethical guidelines, and the ethical approval was obtained from the ethics and review committee from the specialist dental center and research institute (SDCRI-023-20). The teeth extracted for aesthetic enhancements were collected from the patients visiting orthodontic dental clinics of the institute. Only the teeth free from caries, restorations, and other obvious defects were included in our study.

### 2.1. Synthesis of the EA

Following the earlier recommendations of Ye et al. [23], we synthesized our EA. For the preparation of EA, we used a mixture of monomers that included bisphenol A glycol dimethacrylate (BisGMA), triethylene glycol dimethacrylate (TEGDMA), 2-hydroxyethyl methacrylate (HEMA), and ethyl 4-dimethylamino benzoate, and camphorquinone (Esstech Inc., Essington, PA, USA). Our composition entailed 50%-Bis-GMA, 25%-TEGDMA, and 25%-HEMA (60%) by weight with ethanol (30% m/m) used as a solvent. The photo-initiators that encompassed 0.5% (n/n) ethyl 4-dimethylamino benzoate and 0.5% camphorquinone were added in accordance with the monomer moles. Furthermore, we added 1.0% (n/n) diphenyliodonium hexafluorophosphate (DPIHP) as an electron initiator to the adhesive blend. This mixture was formulated inside a triple-necked flask with the help of a magnetic stirrer and a condenser (SA300; Sansyo, Tokyo, Japan). The newly formulated adhesive was isolated in a dark chamber that was shielded with a foil to prevent photopolymerization.

### 2.2. Incorporation of Nano-HA Particles inside the Adhesive

The nano-HA particles (Hydroxyapatite, Sigma Aldrich, MI, USA) were acquired commercially and then silanized. To promote nano-HA particles’ adhesion, 5% silane was incorporated in the 95%-acetone solvent for silanization. The silanized nano-HA particles were incorporated to the EA (10% concentration, m/m) to produce an adhesive containing 10 wt.% HA (HA-10%). To ensure the homogenized dispersion of nano-HA particles inside the adhesive, sonication inside a centrifuge was executed. The synthesized adhesives were deposited for 24 h at 37 °C to permit the evaporation of the solvent. These adhesives were kept safe at 4 °C and were used within 20 days of their formulation.

### 2.3. Characterization of Nano-HA Particles and Adhesives

To analyze the shape of the nano-HA particles, we used the scanning electron microscopy (SEM) technique. To certify the existence of nano-HA particles inside the adhesives, SEM, EDX, and micro-Raman spectroscopy were performed. EDX mapping was used to recognize essential elements inside the adhesives. Before SEM analysis, the HA containing adhesives were positioned on a glass slide and then cured for 20 s with a curing unit (Eliphar S10; 600–800 mW.cm^−2^ output; 3M ESPE, St. Paul, MN, USA). Then, the cured samples were fixed on aluminum stubs and coated with a layer of gold using a sputter coater (Baltec sputter, Scotia, NY, USA). The SEM micrographs were taken at various magnifications based on convenience with 10 kV accelerating voltage using an SEM (FEI Quanta 250, Scanning Electron Microscope, OR, USA). Micro-Raman spectroscopy was performed to characterize nano-HA particles. A Micro-Raman spectrophotometer (ProRaman-L Analyzer; TSI, Shoreview, MN, USA) having software (Raman reader^®^) was used to acquire Raman spectra(s). The laser beam was secured via a 0.9 objective lens, and 600 mW power and 60 s scan were completed three times.

### 2.4. Preparation of Teeth and Bonding Procedure

Ninety teeth (N = 90) were collected, cleaned, and observed under a stereomicroscope (Nikon SMZ800, Japan). Then, these teeth were exposed for 48 h to chloramine trihydrate solution (Merck, Germany). At the level of cementoenamel junction (CEJ), these teeth were embedded in orthodontic resin (Opti-Cryl, South Carolina, Columbia) with 15 mm high sections of polyvinyl pipes (4 mm) and were kept in distilled water (DW). Then, the occlusal enamel was removed with the help of a low-speed cutter (IsoMet^®^ Low-Speed Cutter, Buehler, IL, USA) with a 4-inch diamond blade. The conditioning of dentin was performed utilizing phosphoric acid (H_3_PO_4_) etching, photodynamic therapy (PDT), and ER-CR-YSGG (ECY) laser (Biolase^®^, Waterlase I-Plus, MZ8; Foothill ranch, CA, USA) that formed the basis of our six study groups.

Gp-1 (EA-ER): Dentin surfaces were treated for 15 s with phosphoric acid (H_3_PO_4_, Ivoclar Vivadent-Total etch). This step was followed by their drying, which was performed for 5 s (blot dried to attain a smooth, shiny surface and prevent complete drying–wet bonding). The EA was applied on the dentin surface using a micro-brush for 5 s along with air blowing. The excess EA was removed; then, the remaining EA was photopolymerized (Bluephase^®^, Ivoclar Vivadent, Schaan, Liechtenstein) for 10 s with light having an intensity of 1000 mW/cm^2^.

Gp-2 (EA-PDT): Dentin surfaces were treated with PDT using a 100 mg/L methylene blue (MB) solution (Qualigens Fine Chemicals, Mumbai, India). In order to irradiate the surface, diode laser (ADV Laser, Picasso, Italy) having a monochromatic light (wavelength: 638 nm, power 1.5 W) was used. Keeping perpendicular to the dentin, the fiber optic tip of the laser was positioned in continuous motion for 30 s. With DW, the conditioned dentin surfaces were washed, which was trailed by its air-drying for 5 s. The EA was smeared on the dentin surface and then photo-polymerized (as explained for Gp-1).

Gp-3 (EA-ECY): Dentin surfaces were treated for 60 s with ECY laser (frequency: 30 Hertz, Power 4.5 W). A distance of 2 mm between the laser and dentin was maintained. Post-treatment with the ECY laser, EA was applied on the dentin surface and then photo-polymerized (as explained earlier for Gp-1).

Gp-4 (HA-ER): Dentin surfaces were conditioned with H_3_PO_4_ as described for Gp-1. Post-conditioning, the adhesive HA (HA-10%) was applied on the surface in a similar manner as explained for Gp-1.

Gp-5 (HA-PDT): Dentin surfaces were treated with PDT as enlightened for Gp-2. After conditioning, the HA-10% adhesive was identically applied on the surface as described for Gp-1.

Gp-6 (HA-ECY): Dentin surfaces were treated with ECY laser as explained for Gp-3. After conditioning, the HA-10% adhesive was applied on the surface in a similar way as Gp-1.

All our study groups contained fifteen teeth (*n* = 15). For each tooth, composite resin build-up of 2 mm increments was achieved (Filtek Universal; 3M ESPE) utilizing a resin mold and metal condenser. This incremental build-up was polymerized for 20 s by means of a light-curing unit (Curing Light Eliphar S10; 3M ESPE), and the access material was removed. All these bonded samples were stowed for 24 in DW at 37 °C. Five teeth (of fifteen) in each group were used to evaluate the resin–dentin interface’s bond integrity utilizing SEM, whereas the remaining ten were tested for μTBS investigation.

### 2.5. SEM Investigation of the Bonded Adhesive–Dentin Interface

Bonded beam samples (*n* = 5) from each of the six adhesive groups were polished (Beuhler Polisher, Lake Bluff, IL, USA) and cleaned for 4 min in an ultrasonic bath (Bandelin Digital, Sigma-Aldrich Darmstadt, Germany). The beams were treated for 10 s with 35% phosphoric acid (Ultra etch Econo Kit, Optident, Yorkshire, UK) at the interface, and then, DW was used for washing for 15 s. Then, the samples were immersed for 5 min in sodium hypochlorite (NaOCl) solution having 5.25% concentration and washed. Ethanol treatment at different concentrations (80–100%) was completed to desiccate the samples. Then, these were fixed on aluminum stubs, gold sputter-coated, and analyzed with an SEM (FEI Quanta 250, Scanning Electron Microscope, OR, USA) at an accelerating voltage of 10 kV.

### 2.6. μTBS Testing and Failure Mode Analysis

Bonded samples (*n* = 10) were partitioned using a low-speed cutter (IsoMet^®^ Low-Speed Cutter, Buehler, IL, USA) with a 4-inch diamond blade to create 1 mm × 1 mm composite–dentin bonded beams. Six beams were utilized from each tooth for μTBS analysis (sixty beams in all adhesive groups). These beams were held with the micro-tensile tester (Bisco Inc., Richmond, VA, USA) jaws with the help of cyanoacrylate (Superglue, MN, USA). These were analyzed under tension at a crosshead speed of 0.5 mm/minute until failure. The failure modes were categorized into three types: adhesive, cohesive, or mixed and evaluated with a digital microscope (Hirox KH 7700, Tokyo, Japan).

### 2.7. FTIR Spectroscopy and DC Analysis

FTIR spectroscopy was used to calculate the DC of adhesives (EA and HA-10%). These adhesives were assessed pre- and post-curing. Homogenized adhesives were applied on the potassium bromide disc of the spectroscope (Shimadzu, Kyoto, Japan). While the adhesives were in communication with the sensors of the FTIR (Thermo Scientific Nicolet iS20 FTIR spectrometer, MA USA), the absorbance peaks for C-C double bonds were documented for the unpolymerized resin. After polymerizing the adhesive resins for 40 s with a curing light, the FTIR peaks were observed again. Using the method of Alhenaki et al. [24], C-C aromatic reference peaks (1607 cm^−1^) and C=C (aliphatic) absorbance peaks (1638 cm^−1^) were collected. To determine the DC, FTIR spectra were appreciated between 400 and 4000 cm^−1^. The adhesive’s transformation rates were calculated using the ratios of C=C and C-C absorbance intensities (percentage of unreacted double bonds) pre- and post-photo polymerization using the following equation suggested earlier by Al-Hamdan et al. [8]

DC = [1’−(C aliphatic/C aromatic)/(U aliphatic/U aromatic)] × 100%(1)

where C aliphatic is described as the 1638 cm^−1^ absorption peak of cured resin, C aromatic is the 1607 cm^−1^ absorption peak of cured resin, Ualiphatic is the 1638 cm^−1^ absorption peak of uncured resin, and Uaromatic is the 1607 cm^−1^ absorption peak of uncured resin.

### 2.8. Statistical Analysis

The μTBS and DC analysis mean and standard deviation values were evaluated statistically utilizing SPSS-20.0 (IBM, Chicago, IL, USA). The ANOVA and post-hoc multiple comparison tests were utilized. The significance level was set at 1%.

## 3. Results

### 3.1. SEM, EDX, and Micro-Raman Spectroscopy Results

**Figure 1:**
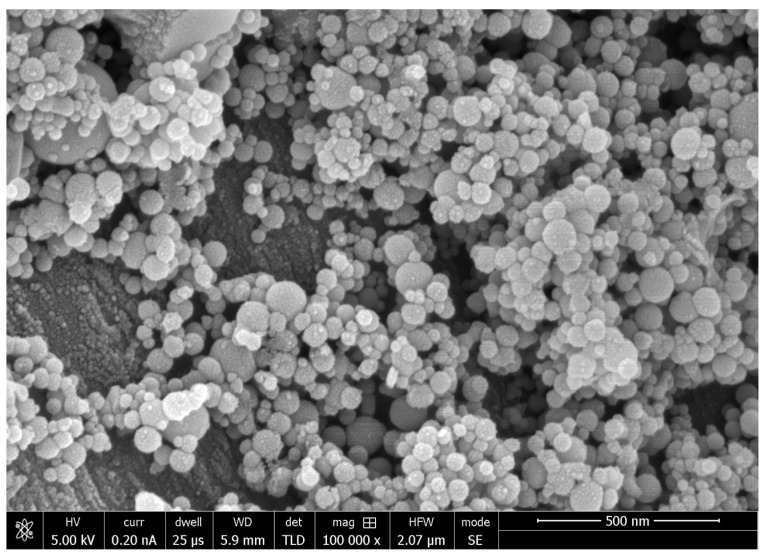
SEM image of HA nanoparticles. These nanoparticles on SEM showed a uniform round shape in mostly agglomerated form with few isolated particles.

**Figure 2:**
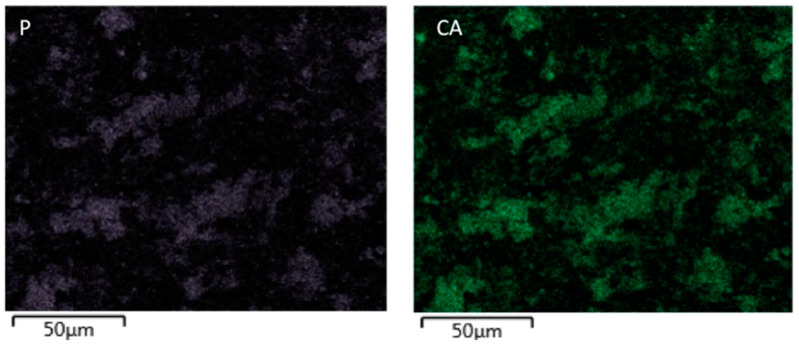
EDX element mapping evaluation of the HA nanoparticles. The two figures show evidence of calcium (**CA**), and phosphorus (**P**) in the samples.

**Figure 3:**
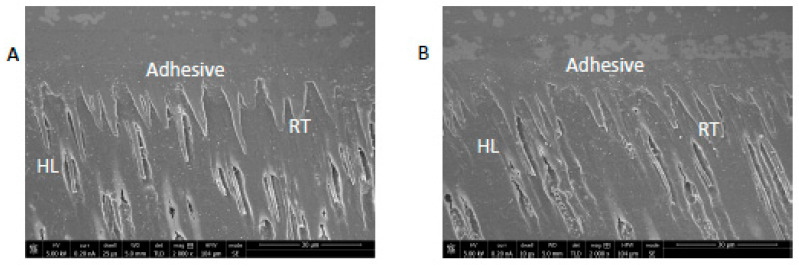

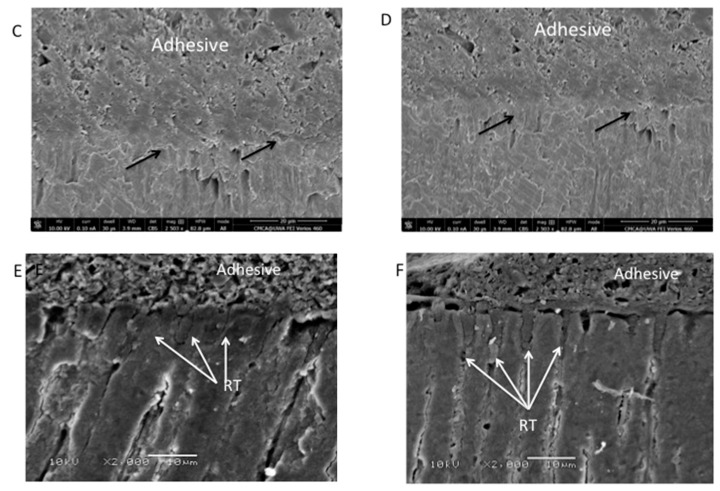
SEM images of bonded dentin specimen. (**A**) HA-PDT group specimens showing standard hybrid layer (HL) formation with low to average resin tag formation in dentin. (**B**) HA-ER group specimens showing classic HL formation with a high number of resin tags. (**C**) HA-ECY group specimens showing uneven dentin surface and craters (arrows) at the interface with no resin tags and dentin penetration. (**D**) EA-ECY group specimens displaying craters (arrows) on the dentin surface at the interface with little to no HL and missing resin tags, showing minimal dentin penetration. (**E**) EA-PDT group specimens showing a low number of resin tags in dentin. (**F**) EA-ER group specimens showing standard HL and multiple resin tag formation.

**Figure 4:**
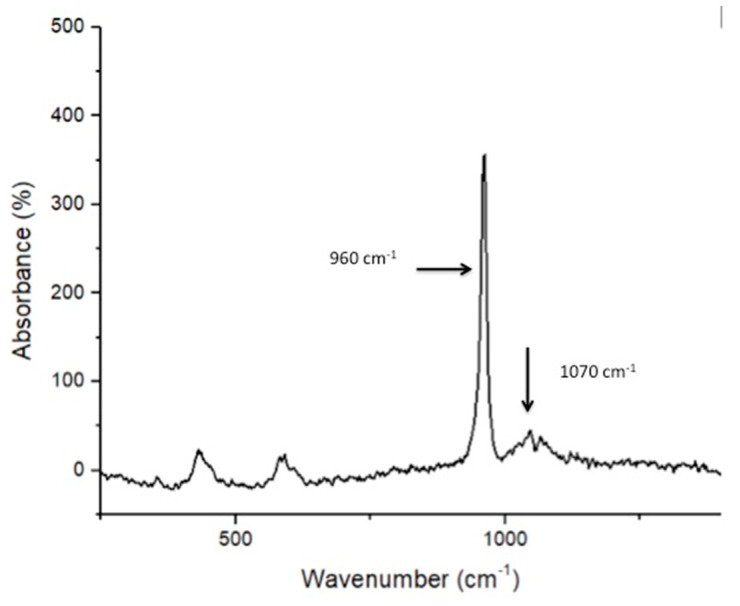
Raman spectra of the HA nanoparticles showing chemical groups identified by Raman analysis that could be identified as phosphate (υ1 PO4 ≈ 960 cm^−1^) and carbonate group at 1070 cm^−1^.

**Figure 5:**
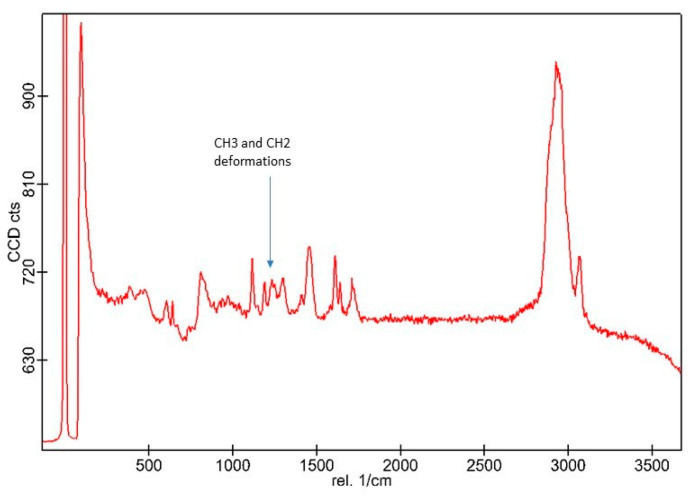
CH3 and –CH2 deformations within the EA are represented by peaks between 1410 and 1460 cm^−1^.

SEM analysis of nano-HA particles revealed spherical particles without any sharp edges, mainly in an agglomerated form (Figure 1). Essential ions (calcium, and phosphorus) were witnessed in the samples on EDX analysis (Figure 2). Both etching and PDT treatments demonstrated hybrid layer development and tag formation for EA (not shown) and HA-10% (Figure 3A—HA-PDT, Figure 3B—HA-ER). In both groups, ECY treatment resulted in no hybrid layer formation and resin tag development (Figure 3C—EA-ECY, Figure 3D—HA-ECY), which was probably due to the damage to the tubular structure of the dentin. Micro-Raman spectra of nano-HA particles revealed the existence of phosphate group at 960 cm^−1^ and carbonate group at 1070 cm^−1^ (Figure 4), which is typical for HA particles. Figure 5 presents the micro-Raman spectra of EA adhesive with peaks between 1410 and 1460 cm^−1^ representing CH_3_ and –CH_2_ deformations within the adhesive.

### 3.2. μTBS Testing and Failure Mode Analysis

The μTBS MPa values (Mean ± SD) detected for both EA and HA-10% groups when they were conditioned with different treatments are shown in Table 1. It can be appreciated that the greatest mean μTBS values were observed for HA-Etch (30.67 ± 3.81) followed by EA-Etch (28.74 ± 4.0). The next greatest values were observed for EA-PDT (27.82 ± 5.51) trailed by HA-PDT (26.10 ± 3.26), whereas the lowest values were appreciated for ECY-treated dentin adhesives (HA-ECY = 15.47± 5.04, EA-ECY = 14.16 ± 4.31). On intra-group comparison, significant differences (*p* < 0.01) were seen when μTBS values of EA-Etch were associated with EA-ECY and when EA-PDT values were matched with EA-ECY. Significant differences (*p* < 0.01) were also detected when HA-Etch values were matched with HA-PDT and HA-ECY and when HA-PDT values were paralleled with HA-ECY (Table 1). None of the inter-group comparisons were statistically significant (*p* > 0.01). No distinct pattern of failure modes was observed in our study, although most of the failures were of the adhesive type (Table 2). Adhesive failures are defined as failures in the adhesion process with accompanying fractures that are not seen in the resin or dentin [25]. The second most common failure seen in our study was the mixed type, whereas the cohesive failure was only observed for HA-PDT.

### 3.3. FTIR and DC Investigation Results

**Figure 6:**
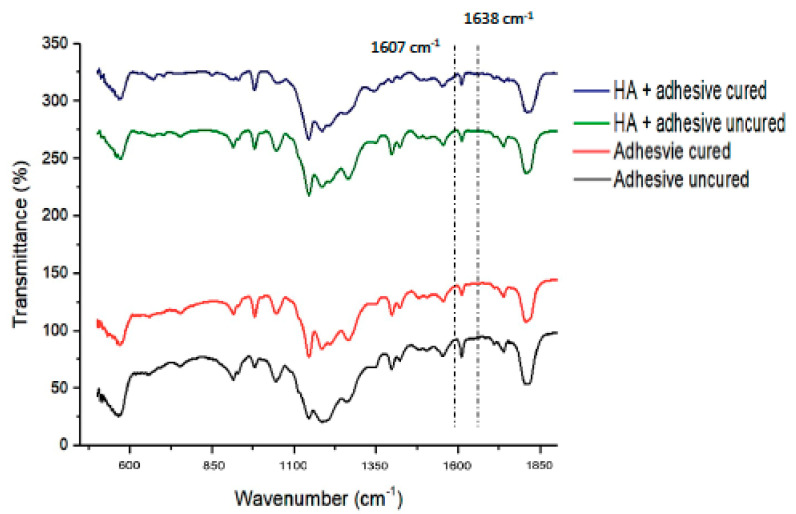
FTIR spectrum of uncured and cured resin adhesives containing HA nanoparticles and control (without nanoparticles). The DC was estimated from 1607 cm^−1^ and 1638 cm^−1^ wavenumber.

The representative FTIR spectra of HA group (cured and uncured) were recorded and merged in Figure 6. The DC was estimated by approximating the disparities in peak height ratio of the absorbance strengths of aliphatic C=C peak at 1638 cm^−1^ and that of a standard inner peak of aromatic C=C at 1608 cm^−1^ while curing, as equated to the uncured adhesive as designated by scattered lines (Figure 6). In terms of DC analysis, the greatest DC was seen for EA (60.4 ± 6.2) followed by HA-10% group (56.8 ± 5.5), as shown in Table 3. No statistically significant results (*p* > 0.01) were witnessed when the DC values of EA and HA-10% were compared with each other.

## 4. Discussion

Our study results have verified that the presence of nano-HA particles inside adhesive could improve its μTBS; however, a lower DC was also observed when the HA-10% group was compared with the EA group. Therefore, based on our findings, we partly accept the hypothesis that the presence of nano-HA particles could expand the adhesive’s mechanical properties. The restoration’s clinical success is extremely dependent on the adhesive properties, as a negotiated resin–dentin bond could result in micro-leakage, secondary caries development, and restoration failure [26]. Nano-inorganic fillers have been utilized to reinforce the mechanical properties of dental composites with great success [27,28,29]. Nano-HA particles contain remineralizing ions (calcium and phosphate) and are highly biocompatible [30,31]. These advantages encouraged us to incorporate nano-HA particles in the adhesive and analyze their bond strength, durability, and dentin interaction.

The nano-HA particles that were acquired for our study were spherical-shaped (Figure 1). The literature shows that spherical-shaped filler particles have a lubricating effect on the material (making it more flowable), whereas amorphous particles could restrict the material’s flow, causing an increased viscosity [32]. The EDX mapping of nano-HA particles revealed the presence of all essential (oxygen) and remineralizing ions (calcium and phosphate) (Figure 2). The presence of calcium and phosphate ensures that the material has remineralizing properties [33], resulting in the formation of an intimate bond between the resin and dentin [8]. Resin tag development demonstrates the flow of adhesive resin in the tubules [32], ensuring optimal mechanical interlocking and the development of a firm hybrid layer [34]. In our study, resin tags of varying depth were observed for the EA and HA-10% group when the dentin was conditioned with H_3_PO_4_ and PDT. For these two conditioning protocols, EA and HA-10% groups revealed resin tag formation, but for ECY-treated dentin, both groups revealed no hybrid layer formation, minimal dentin penetration, and missing resin tags. In general, laser irradiation could create porosities in the surface that could lead to a greater depth of resin tag penetration [35]. Aranha et al. previously demonstrated that the use of a laser resulted in the formation of resin tags that were more pronounced than those produced with other techniques [36]. Our results are in disagreement with their study, as the ECY laser-treated dentin surfaces for both groups (EA and HA-10%) demonstrated no hybrid layer formation and resin tag development. A previous study demonstrated that the use of laser within a specific frequency (5–20 Hertz) could cause an enhanced opening of tubules and the greater penetration of adhesive resin tags [37]. However, increased frequency of the laser (25–30 Hertz) could cause cracking and results in the compromised structural integrity of dentin [38]. Our results are in agreement with this study, as we also used an ECY laser with a higher frequency (30 Hertz) that damaged the dentin, explaining the absence of hybrid layer and lost resin tags. Another plausible reason for our finding that could have contributed to the non-formation of the hybrid layer and resin tags in laser-treated dentin was explained formerly by Ceballo et al., who reported that laser ablation could fuse collagen fibers together, reducing the inter-fibrillar space that is available for the penetration of adhesive [39]. On micro-Raman spectroscopy for nano-HA particles, we observed characteristic peaks for the phosphate group at 960 cm^−1^ and the carbonate group at 1070 cm^−1^. Our Raman spectroscopy findings were in line with several previous studies that also observed peak intensities of phosphate and carbonate groups at 960 cm^−1^ and 1070 cm^−1,^ respectively, for HA-containing materials [6,8,40].

We employed μTBS testing to analyze the bond strength of the two adhesives used in our study after conditioning the dentin. Wagner et al. have previously recommended that the use of inorganic fillers containing a wt.% of >10% could decrease its bond strength due to increased viscosity [41]. Keeping this recommendation in mind, we did not add HA fillers with a concentration greater than 10 wt.%. It was observed from our results that the greatest μTBS values were observed for the HA-10% group on the dentin that was conditioned with H_3_PO_4_, which was followed by the EA group on the dentin again that was conditioned with H_3_PO_4_. In certain previous studies, it was reported that HA adhesives exhibited greater bond strength than the EA, and our results are in agreement with those studies [4,6,8]. Nano-HA-containing materials are able to release calcium and phosphate remineralizing ions effectively [42], and this property could have resulted in improved μTBS values seen in our study for the HA-Etch group. Another plausible reason for this finding could be that the nano-HA particles can biomineralize with the collagen fibers of the dentin [26], causing improved remineralization and enhanced bond integrity, as observed in our study. In addition, this biomineralization could lead to immediate dentin sealing, which potentially increases the bond strength and development of a stress-free dentin bond, as recommended formerly by Sinjari et al. [43]. In our study, μTBS values for both adhesive groups were higher for the dentin conditioned with H_3_PO_4_ than PDT-treated dentin, whose values were higher than ECY conditioned dentin. Previously, Almutairi et al. reported similar findings and demonstrated that the etched dentin presented higher bond strength than PDT and laser-treated dentin [44]. In another earlier study, Alaghehmand et al. revealed that the composite resin applied to the etched dentin demonstrated greater bond strength than the laser-conditioned dentin [45]. Our results are in line with the aforementioned studies, as we also observed the greatest bond strength when the adhesives were smeared on the H_3_PO_4_ etched dentin, as compared with PDT or laser-conditioned dentin. Comparing our μTBS results to the commercial adhesives, it can be seen that our μTBS values for both groups demonstrated comparable results to a previous study, which evaluated the μTBS of six commercial adhesives [46]. This comparison positively attests our rationale of using an EA for this study instead of commercial adhesives.

In the present study, the EA group presented higher DC as compared with the HA-10% group. Previous similar studies have shown that although the addition of filler can increase the adhesive’s bond strength, a decrease in DC is usually also observed [6,47]. One credible reason explaining this finding could be that fillers’ addition could provide a medium that is less penetrable for the curing light and transformation of monomers into polymers, thus producing less DC [8]. Another potential cause for this particular result could be that we used a higher filler concentration (10 wt.%), which could have perhaps increased the viscosity of the material, causing a lower DC [4]; however, we did not investigate this aspect in our study. Comparing our DC results to the commercial adhesives, a previous study reported DC ranging between 70 ± 7 and 79 ± 7 [48], and in comparison, the DC observed for both our study groups (EA and HA-10% gp) was less. A credible reason to explain this finding could be the compositional difference between the monomers and solvents of their study [48] and the present study.

Although the results of our study are promising, they should be interpreted with caution, as we can point out a few limitations of our study. We observed improved µTBS values for the HA-10% group as compared with EA, but a reduced DC was also detected. High DC is warranted for the adhesive’s success, as it prevents the prospect of having a reduced interfacial strength [49]. Future studies using a low filler concentration of nano-HA particles are recommended as they could ensure sufficient polymerization of unreacted monomers, subsequently resulting in a high DC. Another limitation of our study was its in vitro nature. The real in vivo environment is dynamic and offers multiple challenges to the foreign restorative material after its clinical use. These challenges include continuous exposure to saliva, pH fluctuations in the oral cavity due to the intake of various acidic food substances, and temperature variations in the oral environment. Considering all these aspects, future clinical trials of our adhesive could provide varied results when compared with the current findings.

## 5. Conclusions

The presence of nano-HA particles (10 wt.%) in the adhesive increased its bond strength as matched to the EA. The HA-10% adhesive also revealed appropriate dentin interaction and improved resin tag formation (observed on H_3_PO_4_ and PDT conditioned dentin). However, HA-10% adhesive demonstrated decreased DC compared to the EA.

## Figures and Tables

**Table 1:** Showing μTBS of two groups post various conditioning protocols.

	Conditioning					
Adhesive	H_3_PO_4_ Etch	PDT	ECYL	H_3_PO_4_ Etch vs. PDT (*p*-Value)	H_3_PO_4_ Etch vs. ECYL (*p*-Value)	PDT vs. ECYL (*p*-Value)
EA	28.74 ± 4.0	27.82 ± 5.51	14.16 ± 4.31	> 0.01	> 0.01	> 0.01
HA-10%	30.67 ± 3.81	26.10 ± 3.26	15.47 ± 5.04	> 0.01	> 0.01	> 0.01
Inter-group comparison (*p*-value)	>0.01	< 0.01	< 0.01			

H_3_PO_4_: Phosphoric acid, PDT: Photodynamic therapy, ECYL: Er:Cr:YSGG laser, EA: Experimental adhesive, HA: Hydroxyapatite.

**Table 2:** Percentage distribution of interfacial failure types among the study groups.

	Type of Failure		
Study Group	Adhesive	Cohesive	Mixed
EA-Etch	80	0	20
EA-PDT	60	0	40
EA-ECY	100	0	0
HA-10%-Etch	70	0	30
HA-10-%-PDT	70	10	20
HA-10%-ECY	100	0	0

**Table 3:** DC displayed by two adhesive groups.

DC (Mean ± SD) of EA gp	DC (Mean ± SD) of HA-10% gp	*p*-Value
60.4 ± 6.2	56.8 ± 5.5	0.071 *

* Tukey’s test (not significant at *p* ≥ 0.02); EA: Experimental adhesive; HA: Hydroxyapatite.

## Data Availability

Data are available on request from the corresponding author.
